# Convective Drying with the Application of Ultrasonic Pre-Treatment: The Effect of Applied Conditions on the Selected Properties of Dried Apples

**DOI:** 10.3390/foods13233893

**Published:** 2024-12-03

**Authors:** Ewa Jakubczyk, Katarzyna Rybak, Dorota Witrowa-Rajchert, Artur Wiktor, Rafał Rąbkowski, Małgorzata Nowacka

**Affiliations:** Department of Food Engineering and Process Management, Institute of Food Sciences, Warsaw University of Life Sciences, 02-776 Warsaw, Poland; katarzyna_rybak@sggw.edu.pl (K.R.); dorota_witrowa_rajchert@sgggw.edu.pl (D.W.-R.); artur_wiktor@sggw.edu.pl (A.W.);

**Keywords:** air-drying, sonification, drying temperature, physical properties, apples

## Abstract

The aim of this study was to evaluate the effect of ultrasound used as a preliminary treatment and drying temperature on the properties of dried apples (var. Golden Delicious). The aim of the work was also to optimise the process in terms of reducing the drying time and obtaining a product with specific properties. The apple tissue was sonicated for various times from 30 to 60 min. Then, the tissue was air-dried with a constant air flow of 55 to 85 °C. The work determined the dry substance content, water activity, colour parameters, content, antioxidant activity, and hygroscopicity of the dried material. The drying kinetics were also analysed. The results showed that the decrease in sonification time increased the dry matter content and reduced water activity. Also, the decrease in drying temperature caused a smaller intake of water and led to a lower hygroscopicity of dried apples. The selected parameters of the process had a positive effect on the preservation of bioactive compounds and led to an increase in antioxidant activity. Experimental results were adapted by a second-order polynomial model, where analysis of variance was utilized to define optimal drying conditions. Therefore, considering the shortest drying time, the lowest colour difference, ΔE, and the highest antioxidant activity, the best condition for the drying of apple tissue can be obtained with the application of 30 min of samples sonication and drying of apples at a temperature of 80.9 °C.

## 1. Introduction

Ultrasound refers to a range of sound waves with frequencies below those detectable by the human ear. Interest in using ultrasound in food production has remained constant over the years due to the numerous benefits derived from its use. The main advantages of the application of ultrasonic waves for the food industry and consumers are safety, non-toxicity, and eco-friendliness, especially in comparison to other methods that require special use with particular caution, e.g., microwaves, gamma radiation, and pulsed electric fields [1]. Also, the use of sonification may result in intensive mass exchange and shorten the time of many processes, and also reduce energy consumption. It is also a good method to obtain dried herbs, vegetables, and fruits containing a significant amount of health-promoting ingredients [2]. It has been demonstrated that ultrasound-assisted dehydration positively affects the retention of the sensory attributes of the food product, i.e., taste, smell, and colour, and also provides the opportunity to enrich the food with vitamins and minerals. The ease of storing and dosing health-promoting ingredients creates opportunities to design attractive foods from the perspective of a food technologist and consumers [3].

Ultrasound can also be used as a preliminary treatment before the many technological processes. Sonication before osmotic dehydration facilitates mass exchange in the dehydrated raw material by accelerating the removal of water from plant tissues and increasing the dry matter content [4]. Additionally, one of the ways to limit the unfavourable phenomena that occur in the tissue and negatively affect the quality of dried products is to use preliminary treatments before the water removal process, e.g., ultrasound, as a preliminary method before convection drying [5]. The ultrasound pre-treatment of pineapples for 20 and 30 min before convective drying was used. After drying, the US-treated samples had a lighter colour and lower hardness than pineapple slices with sonification. Also, applying treatment for 20 min and 30 min shortened the drying time by 19% and 14.3% [6]. Application of ultrasound pre-treatment before air-drying led to an increase in the process rate and a decrease in the drying time of melon [7], kiwi fruit [8], goldenberry [9], banana, and papaya [10]. Sonification was also applied before the vacuum-freeze-drying of strawberries. US-treated fruits were dried for about 2 h less than the control sample (without pre-treatment). Additionally, the redness, antioxidative substances, and cross-section areas of the matrix in the dried samples with US-treatment were significantly increased [11]. Also, applying ultrasound treatment before the electrohydrodynamic drying of Goji berry [12] and microwave drying of pear [13] positively affected the drying behaviour and different quality attributes of dried fruits.

The sponge effect causes alternating pressure surges, directly influencing the material moisture migration from the material’s deeper layers toward the surface. In turn, the cavitation phenomenon disrupts the laminar layer on the surface of the plant tissue, causing water dispersion or leakage of liquid substances from the raw material. These phenomena can have both a positive and a negative impact on the course of technological processes [14].

Some articles describe the narrow scope of applying ultrasounds as a pre-treatment method before drying. Apples contain many important nutrients and can be a healthy dried snack [15]. The parameters and methods can determine the quality of the finished product. It is also important to reduce energy consumption. It is crucial to select the appropriate sonication conditions to obtain products with tailored properties and quality, especially apples.

The study aimed to determine the effect of ultrasound pre-treatment and the drying temperature on the properties of dried apples. The work aimed to also analyse the process to shorten the drying time and obtain a product with the required properties.

## 2. Materials and Methods

### 2.1. Materials

The apples were supplied by the Experimental Orchard of the Warsaw University of Life Sciences. The winter variety Golden Delicious from one production batch (apples picked in November 2020) was used for the investigation to ensure the repeatability of the results. The collected fruit was stored at a temperature of approximately 4 °C until the tests were performed.

### 2.2. Ultrasonic Pre-Treatment

The apples were cut into 5 mm thick slices and divided into 4 parts. The immersion method of sonication was used. The samples were added to distilled water in beakers in a weight ratio of 1:4. Approximately 130 g of raw material was prepared for each sonication experiment. The process was carried out using an ultrasonic bath MKD Ultrasonic (Warsaw, Poland) with water at room temperature. A bath emitted the waves with a frequency of 21 Hz and power of 180 W for 30, 45, and 60 min. After the treatment, samples (US30, US45, and US60) were dried on filter paper and subjected to further technological processes.

### 2.3. Convection Drying

The raw slices of apple (fresh apples without pre-treatment) and with applied ultrasonic pre-treatment were dried in a convective laboratory drier (Warsaw, Poland) at an air temperatures of 55, 70, and 85 °C and a constant air stream of 2 m/s. The drying equipment consisted of a centrifugal fan blowing air through a heating section (6 coil heaters with 1000 W each) and through a horizontal channel containing the sample on the tray. The air flow was parallel to the material layer. Apple samples were placed in a single layer on a tray with a 2 kg/m^2^ load. The ambient temperature was 22–25 °C and humidity was 50–60%. The POMIAR program (Radwag, Radom, Poland) recorded the mass changes during drying connected with a laboratory microprocessor balance (type A500, Axis, Gdańsk, Poland). Samples were dried to obtain the constant mass (the equilibrium water content). Based on the recorded mass changes during the process, the drying curves of apples were prepared as MR (moisture ratio)–time, and the final drying time was determined as the time at the equilibrium water content.

After drying, the samples were packed in moisture-barrier bags. The obtained samples were subjected to further analyses. The properties of the material subjected to ultrasonic pre-treatment and convection drying (US30-T55, US45-T55, US60-T55, US30-T70, U45-T70, US60-T70, US30-T85, US45-T85, and US60-T85) were compared with reference (control) samples of raw apples after drying at a temperature of 55 (T-55), 70 (T-70), and 85 °C (T-85).

### 2.4. Measurement of Selected Properties of Dried Apples

#### 2.4.1. Dry Matter Content

The dry matter content of dried samples (%) was determined using the oven-drying method at a temperature of 70 °C for 24 h [16]. The measurement was conducted in duplicate.

#### 2.4.2. Water Activity

Water activity was measured with the application of an Aqualab analyser Decagon Devices Inc., Pullman, WA, USA, with a measurement accuracy of ±0.001. The measurements were repeated in triplicate.

#### 2.4.3. Density

The displacement method with hexane to measure the volume of samples was used, according to Nowacka et al. [17]. The weighted samples were poured with hexane into a 25 cm^3^ cylinder. The determination was performed in triplicate. The density was calculated as the ratio of the mass sample to its volume.

#### 2.4.4. Colour

The colour attributes of dried samples were determined in the CIE L*a*b* system using a CR colorimeter (Minolta, Osaka, Japan). The measurement was repeated 10 times. The protocol of measurement and description of colour attributes L, a*b* of dried materials and the total colour change, ΔE, between fresh and dried samples were presented in the work of Jakubczyk and Jaskulska [18].

#### 2.4.5. Hygroscopicity

The hygroscopic properties of dried apples were measured in duplicate according to the procedure applied to dried samples. The dried apples were weighed and stored in glass jars over a NaCl solution with a water activity of 0.75 [18]. After 24 h of storage, samples were weighed. Based on the dry matter content of dried apples and the gain of water vapor, the water content of samples after 24 h was calculated (g H_2_O/100 g d. m.).

#### 2.4.6. Total Polyphenolic Content and Antioxidant Capacity

The content of total polyphenolic compounds was determined using the Folin-Ciocalteu method, with modification [16], in fruits subjected to pre-treatment and convection drying using gallic acid as a standard. An amount of 0.5 g of dried sample was weighed, placed in a beaker, and homogenized with 25 mL of 80% aqueous ethanol. The distilled water, extract, and Folin reagent were stirred, and after 3 min the sodium carbonate solution was added. The obtained solution was stored in the dark for 1 h. The absorbance of supernatant was measured using a Heλios spectrophotometer (Thermo Electron Corporation, Waltham, MA, USA) at a wavelength of 734 nm. The reverence sample with distilled water was prepared as a control. The total phenolic content was expressed in mg of gallic acid equivalents (GAE) per 100 g of dry matter.

The supernatants (extracts) were used to measure the antioxidant capacity with the application of ABTS^•+^ radical. To determine changes in the ABTS^•+^ cation radical, it was incubated with the prepared extract for 6 min. Antioxidant activity was measured spectrophotometrically (Thermo Electron Corporation, Waltham, MA, USA) using a wavelength of 734 nm. The EC50 coefficient described the extract concertation required for a 50% reduction of ABTS^•+^ cation radicals (mg of dry matter per mL of the extract). Analyses of total polyphenolic content and antioxidant capacity were performed in duplicate.

### 2.5. Statistical Methods: Analysis of Variance, Experimental Design

An experiment planning procedure was implemented based on a two-factor experiment at three levels, with the experiments repeated three times at the central point (0.0). According to the assumptions, the test was performed at a constant air flow of 2 m/s, while the pre-treatment time and drying air temperature were optimized by the parameters presented in Table 1. Experimental results were adapted by a second-order polynomial model describing the response variables as a function of independent variables [19].

(1) $$Y=b_{o}+b_{o1}X_{1}+b_{2}X_{12}{+b}_{12}X_{1}X_{2}+b_{1}^{2}X_{1}^{2}+b_{2}^{2}X_{2}^{2}$$

where *Y* is the predicted response; *X* is the independent variables; and *b*_o_, *b*_1_, *b*_2_, *b*_12_, *b*_1_^2^, and *b*_2_^2^ are the coefficients of regressor of the mean, linear, interaction, and quadratic terms.

The response surface methodology (RSM) was also used to optimize the conditions of two factors: the drying temperature with US pre-treatment for selected determinations: drying time, absolute colour difference, and antioxidant activity (EC50); the statistical profiles of approximate values were obtained.

The statistical analysis for the conducted research was performed based on a one-way analysis of variance with a confidence level of α = 0.05. Homogeneous groups were determined using the Tukey HSD Test. Also, two-way ANOVA was used to determine the effect of ultrasonic time and drying temperature on apples’ selected physical and chemical properties.

All analyses were carried out using Statistica software v. 13 (StatSoft Inc., Tulsa, OK, USA).

## 3. Results and Discussion

### 3.1. The Selected Physiochemical and Physical Properties of Apples Dried with and Without Ultrasonic Pre-Treatment

The selected properties after drying of apples with and without ultrasonic pre-treatment were analysed. Table 2 shows that the dry matter content of dried apples ranged from 87.7 to 94.7%. Fresh apples had a dry matter content of 13.9 ± 0.1%. This means that the moisture content of the dried samples has been reduced to a level at which the dried products can be stored. The samples dried at the same drying temperature with pre-treatment and without sonification did not differ significantly. The results of a two-way ANOVA showed that drying temperature was a significant factor that affected the content of dry matter in the samples. The effect of sonification was not significant (Table 3). However, Rodrigues et al. [20] observed an increase in water loss in osmotically dehydrated papaya with the extension of sonification from 10 to 30 min. Fijalkowska et al. [21] observed differences in dry matter in apples after sonification. After drying, samples contained the same amount of water (around 7–8%), which is consistent with our results. The effect of sonification can be related to the structure of plant tissue, the applied frequency of ultrasound, and the treatment time.

Water activity is a key parameter in the evaluation of food safety and quality of products. The reduced water activity enables preserving some physical and chemical properties of dried food during storage [22]. Generally, food products with water activity lower than 0.6 can be considered as microbiologically stable [23]. The water activity of dried apples ranged from 0.161 to 0.303, which indicates microbial safety. The highest water activity was obtained for samples dried at a temperature of 55 °C. The decrease in water activity was related to an increase in drying temperature. Table 3 shows how the drying temperature and the sonification time affected the water activity of dried apples. The result of an applied ultrasound wave is more evident at higher drying temperatures (70 and 85 °C). The increase in sonification time caused the reduction of water activity in dried samples. This can be linked to structural changes in plant tissue. The changes in structure and deformation of cell walls as an effect of sonification were observed by Zhang et al. [11] in dried strawberry chips. The more open structure and higher porosity improved the evaporation of US-treated strawberry samples.

The density of dried samples for most samples obtained at the same drying temperature did not differ. Applying a temperature of 70 °C led to a more significant variation in density values (Table 2). The density of dried apples was reduced from 0.41 to 0.28 g/cm^3^, with an increase in sonification time from 30 min to 60 min. A similar trend of decrease in density after sonification was observed for mushrooms [24] and banana slices [25]. However, the results presented in Table 3 indicate that the drying temperature was the main factor affecting the density of drying material.

Hygroscopic properties of dried material should be controlled because, after absorption or desorption of water vapor from the environment, the properties of the product and its quality can be considerably changed. Also, the increased water content of stored samples may lead to the growth of undesirable microflora and reduce the shelf life of food products [26]. The hygroscopicity of dried apples was described by the measurement of the water content of the sample after 24 h of storage in an environment with a water activity of 0.753. A two-way ANOVA analysis indicates that the drying temperature and sonification time affected the hygroscopic properties of dried apples (Table 3). The effect of sonification was different for apples dried at lower drying temperatures when the hygroscopicity increased with increasing time of US treatment. The opposite trend, with decreased water uptake and longer sonification, was observed for samples dried at 70 °C and 85 °C. This may indicate that the porosity of samples and stiffness of wall cells can be significantly changed, leading to changes in hygroscopicity. Still, longer US exposure and higher drying temperatures are necessary.

### 3.2. The Colour and Antioxidant Attributes of Apples Dried with and Without Ultrasonic Pre-Treatment

The colour of the dried product is a significant indicator of its quality. The consumer negatively evaluates the loss of food colour and its changes during storage [27]. Also, the colour can undergo degradation due to the oxidation of pigments and enzymatic and non-enzymatic browning during drying [28,29]. The lightness, L*, of fresh and dried samples (raw apples) were similar (Table 4). The effect of the sonication time on this parameter was not noticeable. However, applying ultrasound pre-treatment caused the brightening of samples compared to apples dried without sonification. The same trend was observed for US-treated and dried carrots [11].

The intensity of the colour depends on the a* and b* parameters, and the drying time affected their values (Table 3). Cao et al. [30] analysed the effect of US on the colour attributes of dried broccoli florets. Ultrasound treatment caused lower colour changes (L*, a*, and b*) as compared to dried raw samples. The a* values increased with the application of US treatment and drying of apples (Table 4). The value of a* indicates the significant proportion of green colour. This is typical of this variety of apple (Golden Delicious). The increase of a* of dried samples was significant after the long sonification of samples (60 min), which resulted in less green and redder colour. The longer time of sonification and oxidation process can accelerate the browning reaction and the presence of more red shade. The statistical analysis showed that the drying time and US time significantly affected the values of a* (Table 3).

The b* attributes describe the proportion of yellow and blue colour. The positive values of b* indicate the yellowness. The fresh samples had a significantly lower value of b* than the dried samples (Table 4). Also, the longer sonification increased this parameter, especially for higher drying temperatures of 85 °C. The significant effect of sonification time on the b* was confirmed by statistical analysis (Table 3).

The total colour difference between fresh and dried samples, ΔE, was calculated (Table 4). The results of ∆E showed that apple sonification and drying process caused significant changes in colour. However, the statistical analysis showed that drying temperature did not affect this parameter, but the effect of US time was observed. Some authors also noticed that, as the sonification time increased, the ∆E of foods increased significantly [28,31,32].

**Figure 1:**
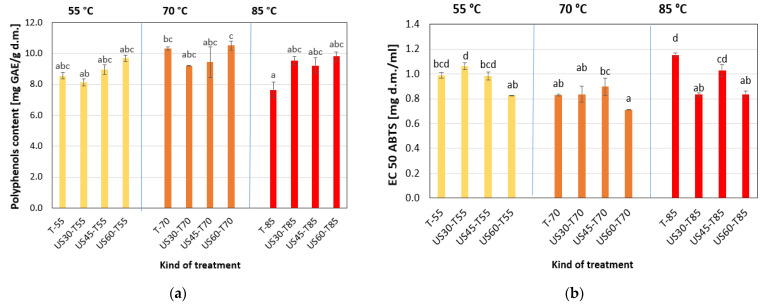
Effect of US pre-treatment and drying temperature on (**a**) EC 50 ABTS and (**b**) Polyphenols content of dried apples. T-55, T-70, T-80: drying temperature of 50, 70, and 80 °C. US30, US45, US60: sonification time of 30, 45, and 60 min. The different letters indicate the significant difference between the values in the bars.

Figure 1 presents the total phenolic content (TPC) of dried apples. The fresh apple contained 7.12 ± 0.13 mg GAE/g d.m.; after drying, this value increased (Figure 1a). The application of US pre-treatment did not cause significant changes in the polyphenols’ content compared to dried apples with sonification. The drying temperature did not affect the parameter’s value, but applying different pre-treatment times was significant (Table 3). A slight increase in the total phenolic content of dried apples was observed with the increase in sonification time (Figure 1a). The same trend was noted by Wang [33] for elecampane. Also, the drying of mushrooms with US application resulted in a better retention of bioactive compounds and higher TPC compared to samples with US treatment [34].

The scavenging activity against EC 50 ABTS^•+^ radical was described by the EC50 coefficient. The lower value of the parameter indicates a higher antioxidant activity. The sonification and drying process of apples caused an increase in antioxidant activity (Figure 1b). Also, statistical analysis showed the significant effect of these two factors on the EC 50 ABTS^•+^ values. Ultrasound exposure reduced the loss in antioxidant properties of red pepper dried at 50 °C but caused higher degradation at 70 °C. The optimal temperature selection during US-treated drying was also important [35].

### 3.3. Drying Kinetics of Apples Dried with and Without Ultrasonic Pre-Treatment

**Figure 2:**
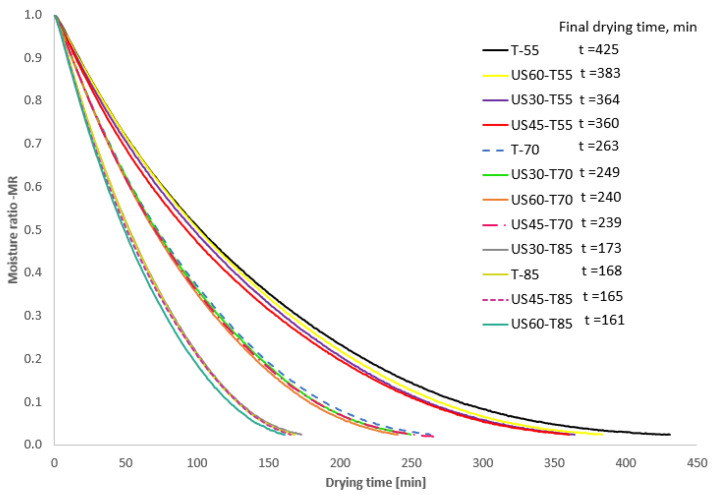
Drying curves of dried apples with and without ultrasonic pre-treatment. T-55, T-70, T-80: drying temperature of 50, 70, and 80 °C. US30, US45, US60: sonification time of 30, 45, and 60 min.

The drying curves of dried apples with and without US treatment are presented in Figure 2. The application of ultrasound led to an increase in drying rate and reduced drying time, with one exception observed for sample U30-T80, which had a longer drying time than for samples without sonification (T-85).

The US pre-treatment caused a decrease in drying time of about 9–15% for fresh apples dried at 55 °C (Figure 2). The sonification and drying at 85 °C reduced drying time by about 2–4% compared to the untreated dried sample. The effect of a decrease in drying time with the application of US with different frequencies and air temperatures was observed for different food products [2,11,20,36]. The intensification of the drying process by the sonification of samples has been explained by the creation of microscopic paths in the material and the weakening of the structure because of the compression and decompression of the material. These accelerate the moisture diffusion during drying [28,37].

### 3.4. Modelling and Selection of the Best Conditions of the Applied Process

**Figure 3:**
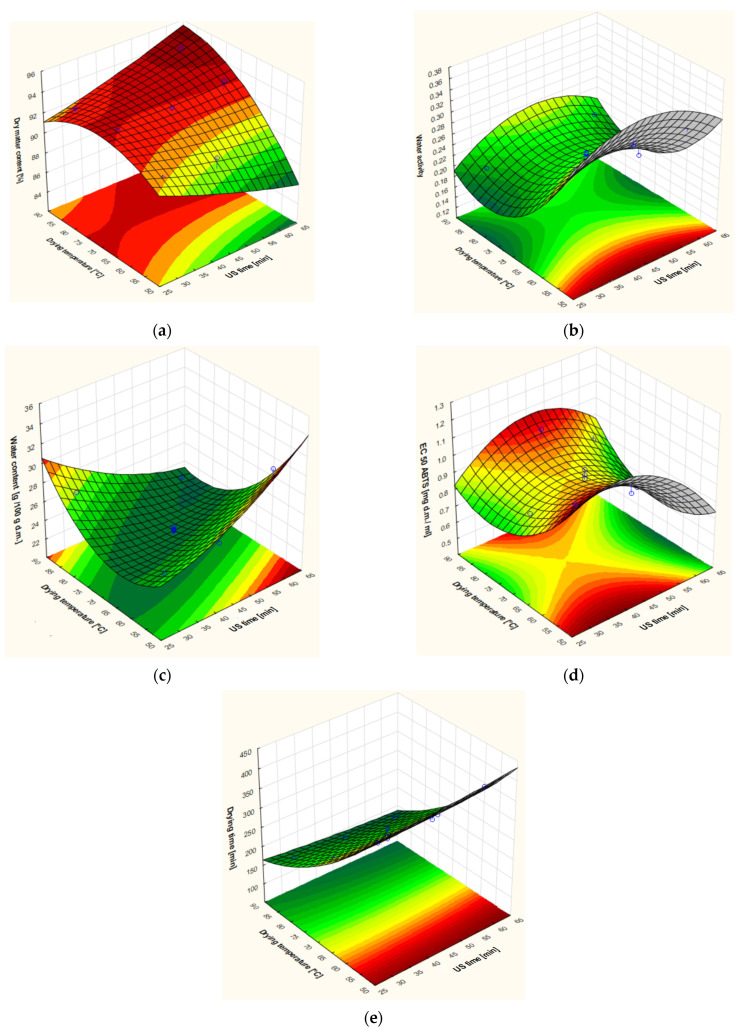
Response surface plots of some selected attributes: (**a**) dry matter; (**b**) water activity, (**c**) hygroscopicity (water content after 24 h of sorption), (**d**) EC_50_ ABTS, (**e**) drying time depending on the drying temperature and time of US pre-treatment of apples.

The results of the second-order polynomial model are presented in Table 5. The response surface for some parameters did not indicate a good model fit. The values of R^2^ and adjusted R^2^ were low for L*, TPC (total phenolic content), and density due to these determinates was not analysed. It was found that the model for dry matter content was well-fitted. The variance analysis showed the significant effect of drying temperature (b_2_): the higher the temperature, the greater the dry matter content (Figure 3a). The dry matter content did not have a significant effect on US time (b_1_), but the interaction term (b_1_b_2_) had a significant effect. In the case of water activity (Figure 3b), the increase in drying temperature caused a decrease in water activity. The US times (b_1_) significantly affected only colour attributes and their values: the increase of sonification time led to an increase in b* and ΔE. Also, changes in water content during the sorption of water during the storage of apples (H24) were affected mainly by drying temperature (Table 5). The surface response plot shows that at a medium drying temperature of 75–80 °C, the lowest value of hygroscopicity can be obtained (Figure 3c). A similar low EC50 ABTS can be obtained in the medium range of drying temperature and time of sonification (Figure 3d). The statistical analysis showed the increase of drying temperature caused the reduction of drying time (Figure 3e).

**Figure 4:**
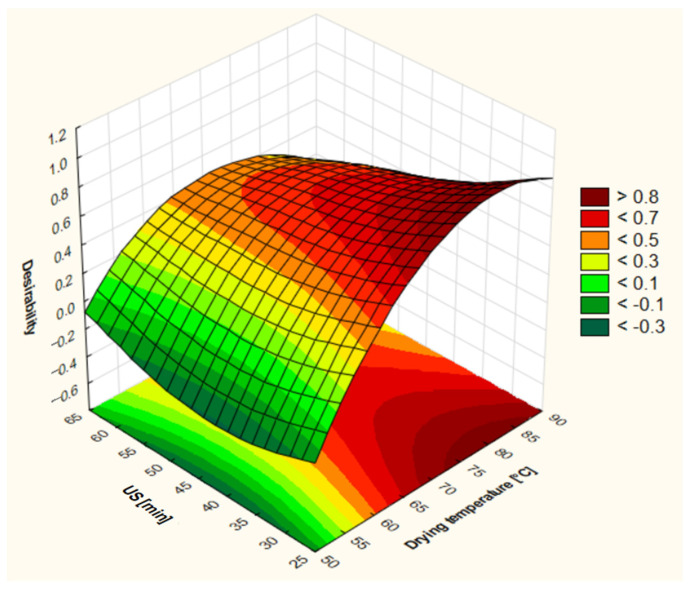
Response plot for desirability of dried apples as a function of drying temperature and time of ultrasonic pre-treatment.

The response surface plot for the desirability of dried apples is presented in Figure 4. All the responses (drying time, EC_50_ABTS, water activity, and ΔE) were optimized to be at a minimum level. Figure 4 represents the overall desirability of a combination of drying temperature and sonification time. Figure 4 indicated that to obtain the highest desirability of 0.896, the optimal conditions were found to be a drying temperature of 80.9 °C and a US time of 30 min. Also, the drying temperature plays a more significant role in combined optimization. Optimum control values for the obtained conditions of drying temperature and US time were 191 min for drying time, 0.831 for EC50ABTS, 0.187 for water activity, and 11.66 for ΔE.

## 4. Conclusions

The applied US-treated and drying temperature enabled the obtained dried products with low dry matter and water activity lower than 0.303, which could guarantee the microbiological stability of stored dried apples. Applying ultrasonic treatment for a longer time led to increased antioxidant activity and phenolic content, which can be linked with a higher extractability of bioactive compounds and a reduction in oxidation rate after sonification. The applied conditions of the apple treatment produced more samples with higher brightness. The application of higher drying temperature reduced drying time, and sonification could accelerate the changes in the structure of apples (deformation of cells). Based on the response analysis results and the optimal minimal values of drying time, EC50ABTS, water activity, and ΔE, the best conditions to obtain these parameters were a drying temperature of 80.9 °C and a sonification time of 30 min.

## Figures and Tables

**Table 1:** Parameters of drying process according to procedure of planning experiments.

Run	Coded Factor X_1_	Coded Factor X_2_	Drying Temperature, °C (X_1_)	US Time, Min (X_2_)
1	−1	−1	55	30
2	−1	1	55	60
3	1	−1	85	30
4	1	1	85	60
5	−1	0	55	45
6	1	0	85	45
7	0	−1	70	30
8	0	1	70	60
9 (C)	0	0	70	45
10 (C)	0	0	70	45
11 (C)	0	0	70	45

**Table 2:** Selected physiochemical and physical properties (dry matter, water activity, density, hygroscopicity) of dried apples.

Kind of Treatment	Dry Matter, %	Water Activity	Density g/cm^3^	Hygroscopicity 24 h, g H_2_O/100 g d. m
T-55	90.5 ± 0.4 ^ab^	0.253 ± 0.003 ^e^	0.44 ± 0.03 ^c^	24.5± 0.3 ^bcd^
US30-T55	91.0 ± 1.5 ^ab^	0.303 ± 0.003 ^f^	0.38 ± 0.00 ^bc^	25.0 ± 0.2 ^cd^
US45-T55	90.7 ± 0.1 ^ab^	0.302 ± 0.002 ^f^	0.38 ± 0.02 ^abc^	25.5 ± 0.2 ^de^
US60-T55	87.7 ± 2.5 ^a^	0.293 ± 0.003 ^f^	0.40 ± 0.01 ^bc^	30.6 ± 0.1 ^g^
T-70	90.6 ± 0.2 ^ab^	0.249 ± 0.002 ^e^	0.45 ± 0.00 ^c^	26.3 ± 0.5 ^ef^
US30-T70	93.0 ± 0.5 ^bc^	0.188 ± 0.004 ^b^	0.41 ± 0.02 ^bc^	23.5 ± 0.5 ^ab^
US45-T70	92.6 ± 0.5 ^bc^	0.234 ± 0.005 ^d^	0.36 ± 0.01 ^abc^	23.8 ± 0.3 ^b^
US60-T70	93.7 ± 0.7 ^bc^	0.189 ± 0.001 ^d^	0.28 ± 0.00 ^a^	22.3 ± 0.4 ^a^
T-85	94.7 ± 1.0 ^c^	0.161 ± 0.002 ^a^	0.36 ± 0.02 ^abc^	24.1 ± 0.9 ^bc^
US30-T85	92.6 ± 1.4 ^bc^	0.200 ± 0.002 ^b^	0.33 ± 0.01 ^ab^	27.2 ± 0.4 ^f^
US45-T85	93.2 ± 0.3 ^bc^	0.224 ± 0.001 ^cd^	0.35 ± 0.00 ^abc^	23.4 ± 0.4 ^ab^
US60-T85	94.6 ± 0.5 ^c^	0.216 ± 0.001 ^c^	0.32± 0.02 ^ab^	23.9 ± 0.3 ^bc^

The different letters indicate the significant difference between the values in the columns. T-55, T-70, T-80: drying temperature of 50, 70, and 80 °C. US30, US45, US60: sonification time of 30, 45, and 60 min.

**Table 3:** Results of two-way variance analysis for selected attributes of dried apples.

Factor	US Time	Drying Temperature
Dry matter	ns	*
Water activity	*	*
Density	ns	*
Hygroscopicity 24 h,	*	*
L*	ns	*
a*	*	*
b*	*	ns
ΔE	*	ns
EC 50 ABTS	*	*
Polyphenols content	*	ns
Drying time	ns	*

ns—not significant effect (*p* > 0.05). *—significant effect (*p* < 0.05).

**Table 4:** Colour attributes of fresh and dried apples.

Kind of Treatment	L*	a*	b*	ΔE
Fresh	75.5 ± 3.2 ^ab^	–3.5 ± 0.4 ^a^	19.1 ± 2.3 ^a^	-
T-55	85.2 ± 3.0 ^b^	–2.6 ± 1.1 ^ab^	31.8 ± 2.1 ^bcd^	16.2 ± 3.1 ^abc^
US30-T55	80.8 ± 4.0 ^ab^	–0.8 ± 1.1 ^bcde^	31.1 ± 2.3 ^bcd^	13.8 ± 2.8 ^ab^
US45-T55	83.3 ± 4.1 ^b^	–2.2 ± 1.1 ^ab^	33.0 ± 3.0 ^bcd^	16.5 ± 2.4 ^abc^
US60-T55	80.0 ± 3.8 ^ab^	0.6 ± 1.1 ^e^	36.3 ± 1.6 ^cd^	18.6 ± 2.0 ^bc^
T-70	81.1 ± 4.3 ^ab^	–1.3 ± 1.1 ^bcde^	33.6 ± 4.1 ^bcd^	16.3 ± 3.8 ^abc^
US30-T70	73.2 ± 3.5 ^a^	–1.5 ± 1.1 ^bcde^	30.4 ± 1.0 ^ab^	12.1 ± 0.9 ^a^
US45-T70	77.7 ± 4.4 ^b^	–0.4 ± 1.1 ^cde^	33.3 ± 2.7 ^bcd^	15.4 ± 3.0 ^abc^
US60-T70	77.5 ± 4.6 ^b^	0.6 ± 1.1 ^e^	37.8 ± 3.5 ^bcd^	19.8 ± 3.5 ^abc^
T-85	83.1 ± 3.6 ^ab^	–1.7 ± 1.1 ^abc^	31.0 ± 4.0 ^bcd^	14.8 ± 3.1 ^ab^
US30-T85	80.9 ± 2.9 ^ab^	–1.7 ± 1.1 ^abcd^	29.2 ± 3.7 ^bcd^	12.0 ± 2.9 ^ab^
US45-T85	79.1 ± 4.8 ^ab^	0.5 ± 1.1 ^de^	30.9 ± 7.6 ^abc^	13.7 ± 7.1 ^ab^
US60-T85	76.6 ± 4.1 ^ab^	2.8 ± 1.1 ^f^	37.8 ± 2.1 ^d^	20.2 ± 2.1 ^c^

The different letters indicate the significant difference between the values in the columns. T-55, T-70, T-80: drying temperature of 50, 70, and 80 °C. US30, US45, US60: sonification time of 30, 45, and 60 min.

**Table 5:** Second-order polynomial results for describing the response variables as a function of independent variables.

	b_o_	b_1_	b_2_	b_1_b_2_	b_1_^2^	b_2_^2^	R^2^	R^2^_adj_
D.m.	77.9	ns	0.618 *	0.006 *	0.001 ^ns^	−0.005 *	0.905	0.825
Aw	1.3	0.009 ^ns^	−0.033 *	0.00003 *	−0.0001 *	0.0002 *	0.947	0.903
L*	140.0	1.041 ^ns^	−2.336 ^ns^	0.004 ^ns^	−0.009 ^ns^	0.017 ^ns^	0.632	0.326
a*	10.3	−0.349 *	−0.189 ^ns^	0.004 ^ns^	0.002 ^ns^	0.0006 ^ns^	0.815	0.661
b*	26.8	0520 *	0.392 ^ns^	0.004 ^ns^	0.005 ^ns^	−0.004 ^ns^	0.919	0.852
ΔE	26.8	0.364 *	−0.208 ^ns^	0.004 ^ns^	0.004 ^ns^	0.00006 ^ns^	0.865	0.753
EC_50_ ABTS	3.4	0.025	−0.084 ^ns^	0.0003 ^ns^	−0.0005 *	0.0005 ^ns^	0.835	0.698
TPC	−5.1	0.010 ^ns^	0.368 ^ns^	−0.001 ^ns^	0.001 ^ns^	−0.002 ^ns^	0.561	0.196
T_drying_	1063	1.609 ^ns^	−14.61 *	−0.03 ^ns^	0.009 ^ns^	0.088 ^ns^	0.990	0.982
Density	0.6	0.004 ^ns^	−0.006 ^ns^	−0.00002 ^ns^	−0.0004 ^ns^	0.00004 ^ns^	0.383	0.100
H24	50.5	0.437 ^ns^	−0.993 *	−0.009 *	0.003 *	0.001 *	0.842	0.709

ns: non-significant, * significant (*p* < 0.05).

## Data Availability

The original contributions presented in the study are included in the article, further inquiries can be directed to the corresponding authors.
